# Fmoc-FF Nanogel-Mediated Delivery of Doxorubicin and Curcumin in Thyroid Cancer Cells

**DOI:** 10.3390/pharmaceutics17020263

**Published:** 2025-02-17

**Authors:** Enrico Gallo, Giovanni Smaldone, Luca Cimmino, Mariantonia Braile, Francesca Maria Orlandella, Neila Luciano, Antonella Accardo, Giuliana Salvatore

**Affiliations:** 1 IRCCS SYNLAB SDN, 80146 Naples, Italy; enrico.gallo@synlab.it (E.G.); luca.cimmino@synlab.it (L.C.); brailemariantonia@gmail.com (M.B.); 2 Dipartimento delle Scienze Mediche, Motorie e del Benessere, Università degli Studi di Napoli “Parthenope”, 80133 Naples, Italy; francescamaria.orlandella@uniparthenope.it (F.M.O.); giuliana.salvatore@uniparthenope.it (G.S.); 3 CEINGE-Biotecnologie Avanzate Franco Salvatore, 80131 Naples, Italy; 4 Dipartimento di Scienze Biomediche Avanzate, Università degli Studi di Napoli “Federico II”, 80131 Naples, Italy; neilaluciano14@gmail.com; 5 Dipartimento di Farmacia, Università degli Studi di Napoli “Federico II”, 80131 Naples, Italy; antonella.accardo@unina.it

**Keywords:** thyroid cancer, nanogel, doxorubicin, curcumin, nanodelivery

## Abstract

**Background:** Thyroid cancer (TC) is the most prevalent endocrine malignancy, and is categorized into well-differentiated and aggressive anaplastic types. Novel therapeutic modalities are needed for TC. Nanomedicine is a promising strategy for the development of precision medicine. In this context, we investigated the use of nanogels (NGs) to deliver agents with different physicochemical properties, specifically the hydrophilic agent doxorubicin (DOX) and the hydrophobic compound curcumin (CUR), in TC cell lines. **Methods:** Nα-9-fluorenylmethoxycarbonyl-diphenylalanine (Fmoc-FF) peptide-based NGs loaded with DOX and CUR were formulated using the solvent-switch method. DOX-loaded NGs were previously characterized. CUR-loaded NGs were characterized through rheology, scanning electron microscopy (SEM), dynamic light scattering (DLS), nanoparticle tracking analysis (NTA), and Fourier transform infrared (FT-IR) spectroscopy. Confocal microscopy, q-RT-PCR, and ATP lite assays were performed to evaluate the uptake and delivery of DOX- and CUR-loaded NGs on TC cell lines. **Results:** CUR-loaded NGs exhibited a mean diameter of approximately 204.3 nm and a zeta potential of −34.6 mV, indicative of a good stability. In vitro release studies revealed a sustained release profile of CUR over 72 h. Functional analyses demonstrated that Fmoc-FF-loaded NGs were internalized into TC cell lines. They were primarily localized in the cytoplasm rather than in early endosomes, thereby ensuring intracellular stability. Furthermore, Fmoc-FF NGs reduced the nuclear uptake kinetics of DOX in TC cells, suggesting a potential reduction in dose-limiting toxicity. Comparative studies with CUR-loaded NGs revealed similar internalization and delayed nuclear uptake, highlighting the efficacy of Fmoc-FF NGs in delivering hydrophobic agents. **Conclusions:** Overall, the data suggest that Fmoc-FF NGs represent a promising strategy for delivering agents with diverse physicochemical properties in TC, enhancing their efficacy and safety and warranting further investigation.

## 1. Introduction

Thyroid cancer (TC) is the most common endocrine-derived neoplasm, encompassing both well-differentiated and undifferentiated tumors. The former are more prevalent and rarely metastatic, while the latter, also known as anaplastic TC, are more aggressive and are associated with a poor prognosis [1,2]. Unfavorable prognostic factors influencing the management of TC patients include age, lymph nodes size, specific mutation events (e.g., BRAF V600E), genetic instability, and intra-tumoral heterogeneity [3,4,5]. Therefore, despite the generally favorable prognosis of well-differentiated TC, novel therapeutic modalities are required for the more aggressive forms.

Doxorubicin (DOX), an anthracycline antibiotic, is used for the treatment of several human neoplasms, including the aggressive forms of TC carcinoma [5,6]. However, despite its potent antitumor activity, the use of DOX is associated with adverse effects, such as cardiotoxicity [7,8]. Therefore, the development of novel delivery systems capable of sending DOX into cancer cells could represent an important strategy for minimizing the toxicity of this agent in TC patients.

Doxil^®^ is a formulation of doxorubicin hydrochloride-loaded PEGylated liposomes. It was the first Food and Drug Administration-approved nanodrug and is currently used in clinical settings for the treatment of breast and ovarian cancers, as well as other solid tumors. Doxil^®^ is a supramolecular drug formulation, capable of enhancing drug localization at the tumor site, by exploiting the enhanced permeability and retention (EPR) effect, while also reducing common side effects, resulting in improved tumor treatment efficacy compared to free DOX [9].

In the field of cancer therapy, natural substances such as fruits, vegetables, tea, and spices have been shown to be beneficial, not only for their antioxidant effects but also as chemosensitizers that improve the efficacy of systemic therapies and reduce their adverse side effects [10,11,12]. Among these, curcumin (CUR), an active hydrophobic natural polyphenol extracted from the rhizome of turmeric, exerts several physiological activities, including antibacterial, anti-inflammatory, and antioxidant effects [13,14,15]. Importantly, CUR demonstrates anticancer properties by reducing the expression of various growth factors and their receptors, cytokines, enzymes, and reactive oxygen species, and also by impairing oncogenic pathways frequently activated during cancer progression, such as TGF-β, AKT/mTOR, and NF-κB [16,17,18]. Furthermore, several studies have highlighted that CUR can enhance the antitumor activity of DOX and reduce its side effects by counteracting inflammation, oxidative stress, and angiogenesis and by inducing apoptosis in cancer cells [19]. Recent studies have demonstrated the effective benefits of CUR in numerous malignancies, including breast [20], liver [21], lung [22], stomach [23], and prostate cancer [24]. Additionally, various clinical trials have been conducted or are ongoing to evaluate the potential use of CUR as an adjunctive treatment in cancer patients [25,26,27,28].

The potential contribution of CUR in TC is well documented by numerous studies that demonstrate its ability to induce cell death, inhibit cell proliferation and motility, and suppress the epithelial-to-mesenchymal transition (EMT) process [29,30]. Interestingly, consistent with findings on other cancers, several lines of evidence suggest that CUR exerts its anticancer effects in TC by targeting and suppressing the activation of the PI3K/AKT [31] and STAT3 [30,31,32] signaling pathways. In TC cell lines, CUR has also been shown to synergistically enhance the anticancer activities of cisplatin and radioiodine treatments [32,33].

However, despite the promising data on CUR, its poor bioavailability and unfavorable pharmacokinetics, attributable to low solubility in water, chemical instability, susceptibility to oxidative degradation, and rapid metabolization, hamper its clinical application [34]. For this reason, novel carrier formulations are necessary to increase CUR concentration in plasma and achieve therapeutic levels in cancer tissues.

Nanomedicines are being designed to enhance drug effectiveness by improving stability, bioavailability, and targeted delivery to cancer cells, thereby minimizing off-target side effects [35,36,37]. In this context, with the advent of nanobiotechnology, nanoparticle (NP)-based drug delivery systems have been proposed and implemented to address these limitations [38]. In addition, to overcome the challenges of traditional chemotherapy, nanoparticle carriers are being explored for the delivery of other anticancer agents, such as non-coding RNAs (e.g., miRNAs) and natural compounds [39,40,41,42,43]. Furthermore, increasing evidence from mouse models and human studies indicates that nanoparticles hold significant promise as diagnostic tools, opening the door to theranostic applications in cancer treatment [44,45,46]. Indeed, NPs offer versatile applications, including drug delivery and theranostic use, due to their tunable physical, chemical, and biological properties [47,48,49].

Among the available nanocarriers, nanogels (NGs) are hydrogel particles ranging in size from 10 to 500 nm, characterized by low toxicity, high hydration, and unique shrinking/swelling properties [48,50]. Additionally, their environmental responsiveness, biocompatibility, and biodegradability make them appealing for pharmaceutical and biomedical applications [51].

In this context, we previously developed a stable peptide-based NG formulation using Fmoc-FF (Fmoc-Phe-Phe-OH, N^α^-9-fluorenylmethoxycarbonyl-diphenylalanine), a low-molecular-weight hydrogelator. This NG formulation demonstrated efficacy in delivering DOX and dexamethasone to breast cancer and leukemic cells, respectively [52,53]. Following this trajectory, in the present study, we evaluated the capability of NGs to deliver both DOX and CUR in TC cell lines.

## 2. Materials and Methods

### 2.1. Reagents

Lyophilized Fmoc-FF powder was purchased from Bachem (Bubendorf, Switzerland). Doxorubicin hydrochloride (DOX), curcumin (CUR) powder, fluorescein-isothiocyanate (FITC), and the surfactants used for NG formulations, i.e., TWEEN^®^60 (polyethylene glycol sorbitan monostearate) and SPAN^®^60 (sorbitan stearate), were purchased from Sigma Aldrich (Milan, Italy). All other chemicals and solvents were procured from Fluka (Bucks, Switzerland) or LabScan (Stillorgan, Dublin, Ireland). Solutions were prepared by weight using doubly distilled water. The concentrations were spectroscopically calculated in solution by UV-vis measurements on a Nanodrop 2000c spectrophotometer (Thermo Fisher Scientific Inc., Wilmington, DE, USA) equipped with a 1.0 cm quartz cuvette (Hellma, Milan, Italy).

The molar absorptivity (ε) values used were as follows: for Fmoc-FF, we used 7800 mol^−1^·L·cm^−1^ at 301 nm; for DOX, we used 9870 mol^−1^·L·cm^−1^ at 480 nm; for FITC, we used 75,000 mol^−1^·L·cm^−1^ at 492 nm; and for CUR dissolved in EtOH, we used 61,864 mol^−1^·L·cm^−1^ at 427 nm.

### 2.2. Formulation of Fmoc-FF Based Hydrogels

Fmoc-FF hydrogels at concentrations of 1.0 wt% were prepared using the “solvent-switch” method [54].

- Formulation of empty Fmoc-FF hydrogel

For the empty Fmoc-FF hydrogel formulation, a Fmoc-FF stock solution in dimethyl sulfoxide (DMSO) at a concentration of 100 mg/mL was diluted 10-fold in water up to a final volume of 300 µL. The metastable suspension, which underwent a transition from opaque to transparent, was allowed to age for 5 min.

- Formulation of DOX-filled hydrogel

The DOX-filled hydrogel (300 µL) was obtained as described above by diluting 30 μL of the peptide stock solution with 270 µL of an aqueous DOX solution at a concentration of 1.14 × 10^−2^ mol L^−1^.

- Formulation of FITC-filled and CUR-filled hydrogels

FITC-filled and CUR-filled hydrogels (300 μL) were prepared using a slightly modified two-step method. In the first step, 15 μL of Fmoc-FF stock solution (200 mg/mL) in DMSO was mixed with 15 μL of either a 1.28 × 10^−2^ mol·L^−1^ FITC solution in DMSO or a 5.43 × 10^−1^ mol·L^−1^ CUR solution in DMSO. In the second step, these mixtures were diluted with 270 μL of double-distilled water and vortexed for 7 s to achieve a homogeneous solution. The suspensions were allowed to age at room temperature for a few minutes.

We assessed the macroscopic formation of all self-supporting hydrogels using the inverted test tube assay.

### 2.3. Formulation of Filled Nanogels

All Fmoc-FF based NGs were formulated from corresponding hydrogels through a top-down submicronization strategy, as previously described [54,55]. In brief, each Fmoc-FF hydrogel was homogenized at 35,000 min^−1^ for 5 min into 1.2 mL of an aqueous solution containing TWEEN^®^60/SPAN^®^60 at a *w*/*w* ratio of 52/48 (total of 1.06 × 10^−5^ mol), using a MICCRA GmbH D9 homogenizer (Buggingen, Germany). The suspension obtained was subsequently tip-sonicated for 5 min at 9 W using a Branson SFX250 homogenizer (St. Louis, MO, USA) [52,55]. The purification of NG formulations was achieved by performing size exclusion chromatography (SEC) on a pre-packed column Sephadex G-50 pre-equilibrated with water.

### 2.4. CUR-Filled Hydrogels and Nanogels Characterization

#### 2.4.1. Rheological Studies

The rheological properties of both empty and CUR-filled hydrogels (HGs) were assessed at 25 °C using a rotationally controlled stress rheometer (Malvern Kinexus, Grovewood Road, UK) with a 15.0 mm flat-plate geometry (PU20:PL61). For each experiment, a freshly prepared HG sample (360 μL) at a concentration of 1.0 wt% was developed and placed in a humidity chamber. A gap of 1.0 mm was used throughout the analyses. To determine the linear viscoelasticity regime, preliminary strain sweeps (0.01–100%) and oscillatory frequency sweeps (0.01–100 Hz) were conducted. Time-sweep oscillatory tests (at 0.1% strain and 1.0 Hz frequency) were performed over a duration of 20 min. Final results are presented as the G′ (storage elastic modulus)/G″ (shear loss or viscous modulus) ratio, expressed in Pascal [Pa].

#### 2.4.2. Scanning Electron Microscopy (SEM)

SEM analyses were performed on CUR-filled HG. Empty and CUR-filled HGs were prepared, placed on aluminum cover slips, and left to air-dry overnight under ambient conditions. The resulting xerogels were then coated with a Au film and imaged using a SEM (JEOL, Tokyo, Japan) operating at 20 kV.

#### 2.4.3. Dynamic Light Scattering (DLS) and Nanoparticles Tracking Analysis (NTA)

The hydrodynamic radii (RH) and the zeta potential (ζ) of all filled NGs were acquired using DLS using a Zetasizer Nano ZS (Malvern Instruments, Westborough, MA, USA). Measurements were conducted at room temperature with a backscatter detector set to 173° in automatic mode, employing disposable calibration cuvettes.

NTA measurements were carried out using a NanoSight NS300 (Alfatest, Italy). All filled NG formulations were diluted 10,000-fold to a final volume of 1 mL with double-distilled water. Dilutions were performed to achieve the optimal particle per frame value (20–100 particles/frame). The parameters were fixed on the basis of the manufacturer’s software manual (NanoSight NS300 User Manual, MAN0541-01-EN-00, 2017). Each measurement was performed in triplicate, and average values were calculated.

#### 2.4.4. Fourier Transform Infrared (FT-IR) Spectroscopy

FT-IR measurements were performed on CUR-filled and empty HGs and NGs using a Jasco FT/IR 4100 spectrometer (Easton, MD, USA) in attenuated total reflection (ATR) mode, equipped with a germanium (Ge) single-crystal element, at a spectral resolution of 4 cm^−1^. Data processing was carried out with integrated software. The spectra were acquired over 300 scans at a scanning speed of 2 mm·s^−1^, using a potassium bromide (KBr) background as reference.

### 2.5. Curcumin Encapsulation and Release from Nanogels

The release of CUR from the NGs was evaluated following preparation and purification to remove free CUR. For the release assay, 1.0 mL of CUR-loaded NGs was suspended in 9.0 mL of 0.100 mol/L PBS. The solution was stirred at 37 °C, and aliquots of 400 µL were collected at fixed time points over a period of up to 72 h. Each aliquot was replaced with an equal volume of fresh PBS to maintain constant conditions.

Purification of NGs from free CUR was carried out using SEC on a pre-packed Sephadex G-50 column pre-equilibrated with PBS. The percentage of released CUR was determined by lyophilizing the samples, dissolving the freeze-dried powder in methanol, and subtracting the amount of free CUR from the total initial CUR loaded into the NGs. We quantified the drug concentration at λ = 427 nm using UV–vis spectroscopy with a calibration curve. We determined the drug loading content (DLC), defined as the ratio of encapsulated CUR (g) to the total weight of surfactant and peptide (g), and the encapsulation ratio (ER%), expressed as the percentage of CUR encapsulated within the NGs relative to the total CUR used during formulation, according to the following formulas:

(1) $$DLC\%=\frac{weight\;of\;encapsulated\;CUR\;(g)}{weight\;of\;peptide\;and\;surfactants\;(g)}\cdot 100$$

(2) $$ER\%=\frac{weight\;of\;encapsulated\;CUR\;in\;NGs\;(g)}{weight\;of\;the\;CUR\;in\;the\;loading\;solution\;(g)}\cdot 100$$

The amount of encapsulated CUR (g) was calculated as the difference between the total CUR added during the preparation process and the fraction of free CUR. The release experiment was conducted in triplicate, and the CUR release profile was expressed as the percentage of drug released relative to the total drug initially loaded into the NGs.

### 2.6. Cell Cultures

The human TC cell lines (K1 and CAL62) were cultured at 37 °C in a humidified atmosphere with 5% CO_2_ using Dulbecco’s Modified Eagle Medium (DMEM) supplemented with 10% fetal bovine serum (FBS), L-glutamine, and penicillin/streptomycin (Thermo Fisher Scientific, Waltham, MA, USA). The CAL62 cell line was obtained from the Deutsche Sammlung von Mikroorganismen und Zellkulturen GmbH (DSMZ, Braunschweig, Germany), while the K1 cell line was acquired from the European Collection of Authenticated Cell Cultures (ECACC, Sigma-Aldrich, St. Louis, MO, USA).

### 2.7. Total RNA Extraction, Reverse and q-RT-PCR

Total RNA was extracted using the TRIzol reagent (Catalog No. 15596018, Thermo Fisher Scientific) following the manufacturer’s instructions and quantified with a NanoDrop spectrophotometer (Thermo Fisher Scientific). Subsequently, 1 µg of total RNA was reverse transcribed into cDNA using the QuantiTect Reverse Transcription Kit (Catalog No. 205313, Qiagen, Hilden, Germany) according to the manufacturer’s protocol.

Quantitative real-time PCR (qRT-PCR) was conducted using the iQ™ SYBR Green Supermix (Catalog No. 1708882, BioRad, Hercules, CA, USA). The thermal cycler program consisted of an initial step at 95 °C for 3 min for polymerase activation and DNA denaturation, followed by 40 cycles of 10 s at 95 °C (denaturation), 30 s at 60 °C (annealing), and 30 s at 72 °C (extension), as per the protocol.

The forward (fw) and reverse (rev) primers used were:

- β-Actin: rev 5′-CCAACCGCGAGAAGATGA-3′; fw 5′-CCAGAGGCGTACAGGGATAG-3′;
- Caveolin 1: rev 5′-GCAGGAAAGAGAGAATGGCG-3′; fw 5′-CGAGAAGCAAGTGTACGACG-3′.

β-Actin was used as a housekeeping gene to normalize Ct (cycle threshold) values of Caveolin 1 gene. The changes in Caveolin 1 mRNA were expressed as 2^−ΔΔCt^.

### 2.8. Confocal Microscopy

FITC, DOX and CUR-filled NGs were added to 20 × 10^3^ K1 and CAL62 cell lines in µ-Slide 8 Well high Glass Bottom (Catalog No. 80806, purchased from ibidi GmbH, Gräfelfing, Germany) in complete medium. The cells were incubated at 37 °C in a humidified atmosphere containing 5% CO_2_ for 24 h. For staining experiments, cells were fixed with 4% paraformaldehyde, permeabilized, and stained with a 1:20 dilution of anti-EEA1 antibody (E9Q6G, Cell Signaling, Danvers, MA, USA) and Alexa647-conjugated secondary anti-mouse antibody (A-21235, Thermo Fisher, USA). Alternatively, cytoskeleton staining was performed using a 1:5000 dilution of Phalloidin (13054S, Cell Signaling, USA). Nuclei were stained with Hoechst 33342, diluted 1:10,000 (Catalog No. 62249, Thermo Fisher Scientific).

### 2.9. Viability Assays

To measure cell viability, the ATPlite Luminescence Assay System (Catalog No. 6016731, purchased from PerkinElmer, Waltham, MA, USA) was used according to the manufacturer’s instructions.

Briefly, 1 × 10^3^ TC cell lines (K1, CAL62) were seeded (100 µL/well) in a 96-well plate and cultured in complete medium at 37 °C in a humidified atmosphere containing 5% CO_2_. The following day, cells were treated with empty, DOX-filled, and CUR-filled NGs, and with free DOX and free CUR for 24, 48, and 72 h. To perform the ATPlite Luminescence Assay, reconstituted substrate (100 µL) was added to each well. After sealing and mixing, luminescence was detected using the Victor 3 Model 1420-012 Multi-label Microplate Reader (PerkinElmer, Waltham, MA, USA).

### 2.10. Statistical Analyses

Data obtained from functional analysis were reported as means and standard error of the mean (±SEM) and elaborated with the GraphPad Prism 9 software (La Jolla, CA, USA). *p*-values less than 0.05 were considered to be statistically significant.

## 3. Results

### 3.1. Formulation and Structural Characterization of CUR-Filled Hydrogels

**Figure 1:**
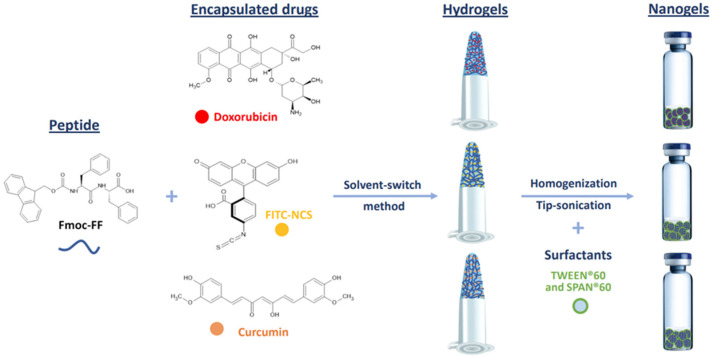
Schematic representation of components and methodologies for the formulation of Fmoc-FF nanogels encapsulating the exploited drugs. Chemical formulae of Fmoc-FF peptide and of doxorubicin, FITC-NCS and curcumin molecules are also represented.

Macroscopic hydrogels (HGs) of Fmoc-FF loaded with DOX, CUR, or FITC-NCS were formulated using the solvent-switch method [54]. Due to the low water solubility of CUR, this drug was dissolved in DMSO (200 mg/mL) rather than water and mixed in an equal volume (15 µL) with the solution of the low-molecular-weight hydrogelator (200 mg/mL) in DMSO. The resulting solution was then diluted 10-fold with water to achieve a final concentration of 1.0% wt. The respective nanogels were prepared by homogenization and sonications (Figure 1).

**Figure 2:**
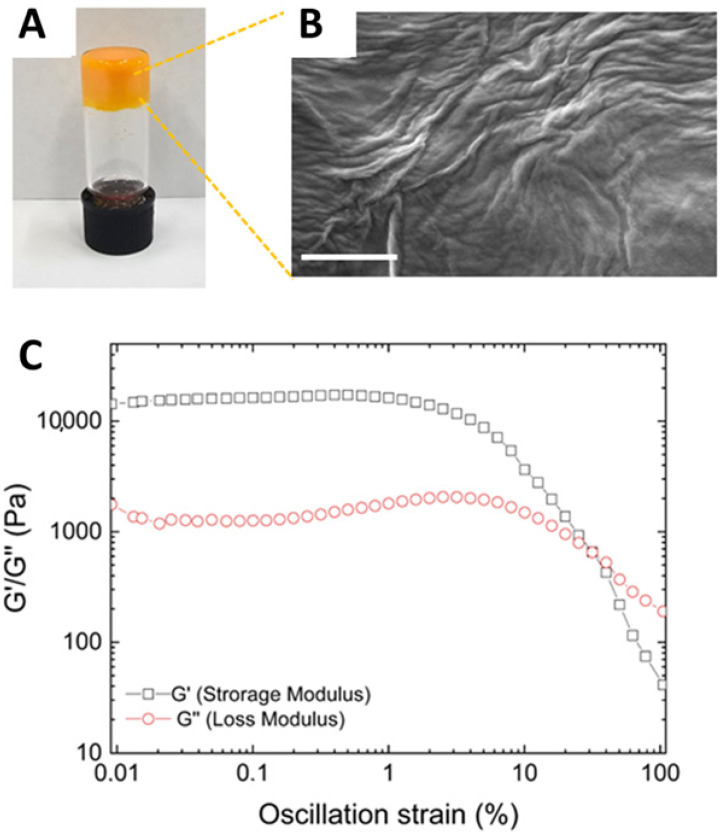
Structural and rheological characterization of CUR-filled hydrogel. (**A**) Inverted tube test. (**B**) Selected microphoto of CUR-filled xerogel. Scale bar: 500 nm. (**C**) Rheological profiles of time-sweep measurements. G′ and G″ are sketched in gray and red, respectively.

As expected for all HGs prepared using this method [53], also the CUR-loaded HG formed as a result of an opaque-to-clear transition (Figure 2A). However, the CUR-filled HG exhibited significantly slower gelation kinetics compared to the empty Fmoc-FF HG (1 h versus 2 min). This slower kinetics is likely attributed to the non-covalent interactions between the peptide matrix and the drug, which participate in the fibril formation. The effective gel state and the absence of syneresis phenomena were assessed using the inverted test tube method (Figure 2A).

The CUR-filled HG was analyzed from the structural point of view through SEM (Figure 2B), FT-IR spectroscopy (Supplementary Materials, Figure S1), and rheological analysis (Figure 2C). The morphological properties of CUR-filled HG were examined via SEM. Micrographs were captured from xerogel samples drop-casted on aluminum stubs (10 µL). An interwoven fibrous network, typically observed in Fmoc-FF HG, was identified in a similar way, as previously reported [53]. Figure 2B also reveals alterations in the HG structure, attributable to the incorporation of CUR, indicating successful drug encapsulation. This consideration aligns with the observation of the gel state of the CUR-filled HG using the inverted test tube method (Figure 2A).

The secondary structure of CUR-filled HGs was analyzed using FT-IR spectroscopy and compared to the structure of the corresponding empty HG (Supplementary Materials, Figure S1A).

Curcumin (CUR) exhibited characteristic absorption peaks at 3508 cm^−1^ (phenolic O-H stretching), 1628 cm^−1^ (aromatic C=C stretching), 1597 cm^−1^ (benzene ring stretching), 1509 cm^−1^ (C=O and C=C vibrations), 1428 cm^−1^ (olefinic C-H bending), 1278 cm^−1^ (aromatic C-O stretching), and 1024 cm^−1^ (C-O-C stretching). For CUR-loaded HGs, peaks were observed at 1627 cm^−1^ (aromatic C=C stretching), 1589 cm^−1^ (benzene ring stretching), and 1516 cm^−1^ (C=O and C=C vibrations). These peaks correspond to curcumin’s signature absorptions, confirming its incorporation into the hydrogel. The weakening and shifting of curcumin’s primary absorption bands upon encapsulation indicate its integration within the hydrogel matrix, where its spectral characteristics were partially masked [56].

Regardless of the sample type, all HG systems demonstrated a shared transmittance profile. No significant differences, apart from intensity variations, were observed between loaded and unloaded HGs. This consistency underscores the role of Fmoc-FF as the common supramolecular structuring element across all systems. Furthermore, FT-IR analysis revealed that curcumin encapsulation at the tested concentrations did not disrupt the overall peptide organization.

For peptide-based aggregates in aqueous environments, FT-IR spectra are predominantly characterized by two key regions: a broad signal in the amide A region (3700–3000 cm^−1^) centered around 3400 cm^−1^ and a sharper band in the amide I region (1700–1600 cm^−1^) near 1641 cm^−1^. The amide A region arises from symmetric and asymmetric stretching of O-H and N-H groups, reflecting the water exposure of the aggregates. Deconvolution of this region, aimed at distinguishing between H-bonded and free N-H groups, did not resolve the individual contributions. The amide I band at 1641 cm^−1^, associated with carbonyl stretching, further indicates the prevalence of β-sheet-rich assemblies.

To mechanically characterize the CUR-filled HG, a rotational rheological analysis was conducted to investigate the viscoelastic properties of the CUR-containing matrix. An initial assessment of the optimal measurement conditions was performed through dynamic oscillation strain sweep (at a frequency of 1.0 Hz) and dynamic frequency sweep (at ω = 0.1% strain), with all data presented as G′ (storage modulus) and G″ (loss modulus). The linear viscoelastic region (LVR) was identified within the range of 0.01 < ω < 4.8%. Time-sweep oscillatory measurements (20 min, 1.0 Hz, 0.1% strain, Figure 2C) were then carried out on CUR-filled HG. As evidenced by the values of G′ (11,800 KPa) > G″ (1075 KPa), it is clear that the sample is in a gel state. The tanδ (G′/G″ = 20.4) further indicates the pronounced viscoelastic nature of the gel, reflecting the formation of a highly robust matrix. The G′ value of the Fmoc-FF HG matrix is notably enhanced by the incorporation of CUR, which is likely attributed to the strong interactions between the hydrophobic drug and the peptide. This finding is consistent with previous observations related to the encapsulation of other highly hydrophobic drugs [53].

### 3.2. Formulation and Structural Characterization of CUR-Filled Nanogels

**Figure 3:**
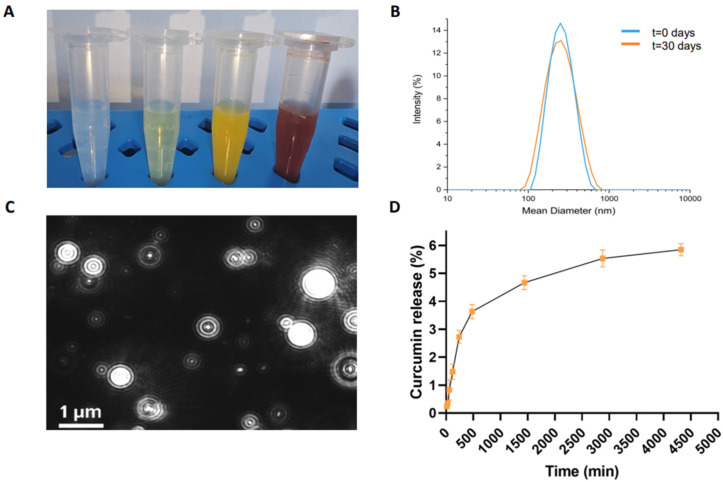
Structural characterization of CUR-filled NGs. (**A**) Images of empty NGs, FITC-filled, CUR-filled, and DOX-filled NGs, from left to right, respectively; (**B**) intensity profile of CUR-filled NG measured by DLS technique at 0 days and after 30 days; (**C**) representative video frame of CUR-filled NG recorded by NTA; (**D**) drug release (%) profile for CUR-filled NG at pH 7.4. Error bars represent the standard error of the mean of three independent experiments.

The corresponding injectable NG formulation was obtained using the top-down method, involving the homogenization and tip sonication of the HG. To stabilize the hydrogel nanoparticles, submicronization was performed in the presence of two biocompatible surfactants (TWEEN^®^60 and SPAN^®^60). The resulting formulation appeared opaque and orange (Figure 3A).

CUR-filled NGs were purified from excess free drug by gel filtration. The amount of encapsulated drug (1.82 mg/mL) was determined by UV-vis spectroscopy. We measured the absorbance at 427 nm of the free CUR, which was lyophilized and then resuspended in EtOH. This amount corresponds to an encapsulation ratio (ER%) of 90.8%, which was significantly higher than the water solubility of CUR (0.6 µg/mL). Representative images of empty, FITC-filled, and DOX-filled NGs are also shown in Figure 3A for comparison.

As previously described for the hydrogels, the CUR encapsulation into the nanogels was further confirmed by FT-IR spectroscopy (Supplementary Materials, Figure S1B).

CUR-filled NGs were analyzed using dynamic light scattering (DLS) and Nanotracking Analysis (NTA), two complementary techniques that provide crucial insights into the size, distribution, and stability of nanoparticles in a suspension.

The DLS intensity profiles (Figure 3B) and the zeta potential distribution revealed nanoparticles with an average diameter of 204.3  ±  80.0 nm, a zeta potential (ζ) of −34.6  ±  1.2 mV, and a polydispersity index (PDI) of 0.167, suggesting a relatively narrow size distribution, which is essential for ensuring the uniform delivery of the therapeutic agent. The samples were measured at a concentration of approximately 2.75 × 10^10^ nanoparticles mL^−1^, at 25 °C. Importantly, no significant changes in the size or surface charge of the nanoparticles were observed after 30 days, demonstrating the long-term stability of the formulation. The absence of nanoparticle aggregation under physiological conditions over time further supports the reliability and suitability of the CUR-filled NGs for extended use in biological environments.

CUR-filled NGs were further characterized using NTA, which confirmed the size distribution of individual particles as well as the particle concentration in the solution.

Figure 3C and Figure S2C in Supplementary Materials shows that the majority of the nanoparticles have a diameter of around 97 nm, with only a minority exhibiting larger sizes, approximately 127 nm and 174 nm. Notably, 90% of the particle size distribution falls below 187.1 ± 12.3 nm, which is consistent with the Gaussian distribution obtained from the DLS profile (Figure 3B). This consistency between DLS and NTA data underscores the accuracy and reliability of the characterization methods employed. NTA profiles of empty, FITC-filled, and DOX-loaded NGs are also shown for comparison (Supplementary Figure S2A, S2B, and S2D, respectively).

A syringeability test was conducted on Fmoc-FF empty NGs as well as CUR- and DOX-filled ones (Supplementary Materials, Figure S3A, S3B, and S3C, respectively). The NGs demonstrated no resistance to syringeability. This confirmed their injectability, which is due to their solution-like nature. Immediately after passing through the syringe, the particle size was again analyzed using DLS. As shown in Supplementary Materials, Figure S3D, no changes were observed in the original size of the nanogels. Furthermore, the analysis of the complex viscosity spectrum, obtained as a function of frequency (Supplementary Materials, Figure S3E), revealed a slight increase in viscosity for the drug-loaded nanogels compared to the unloaded system. This increase in formulation viscosity may contribute to a slower drug diffusion rate from the gel matrix into the surrounding medium.

Table 1 presents the structural data for all the tested nanogel formulations.

### 3.3. CUR Release from Nanogels

The in vitro release of CUR from the loaded NG formulation was evaluated over a 72 h period under controlled conditions. The experiment was conducted in a 0.100 mol/L phosphate-buffer solution (PBS), which was maintained at a constant temperature of 37 °C in a sealed glass tube to minimize solvent evaporation, with gentle stirring performed to facilitate the development of a uniform drug distribution throughout the solution.

After the preparation, purification, and quantification of the encapsulated CUR, 1 mL of the NG suspension was carefully added to the PBS. The incubation took place at 37 °C to mimic physiological conditions. The release of CUR was monitored at specific time intervals over the course of 72 h. The amount of CUR released was measured using UV–vis spectroscopy at an absorbance wavelength of λ = 427 nm, which corresponds to the characteristic absorbance of CUR in the solution.

To quantify the extent of drug release, the data were expressed as a percentage ratio, comparing the amount of CUR released to the total amount initially loaded into the NGs. Figure 3D illustrates the release kinetics from the NGs, with a relatively low cumulative release rate of approximately 5.7% after 72 h. The drug release mechanism of curcumin from Fmoc-FF nanogels may involve a combination of diffusion and swelling processes. When exposed to PBS, the nanogel swells as a result of interactions with water or ions, which facilitates the diffusion of curcumin across the gel matrix. This slow and controlled release profile is indicative of a sustained drug release system, which is highly beneficial for therapeutic applications requiring prolonged drug exposure. The exceptionally slow release observed in this study can be attributed to the strong interaction between the Fmoc-FF peptide and the CUR within the hydrogel matrix. These interactions likely form a complex that not only encapsulates the drug efficiently but also slows down its release by hindering the free diffusion of CUR from the matrix. This behavior is consistent with what is commonly observed in peptide-based drug delivery systems, where the peptide–drug interactions play a critical role in controlling drug release rates.

The prolonged release kinetics of CUR from the NGs suggests that the incorporation of CUR into the NG formulation could provide significant advantages for sustained and prolonged drug delivery. This controlled release could reduce the frequency of drug administration, improving patient compliance and reducing the potential for side effects associated with rapid drug release.

Moreover, these results are consistent with previously published studies, which have shown that similar drug delivery systems based on peptide hydrogels or nanogels can effectively prolong drug release and improve therapeutic efficacy [57,58]. The sustained release of CUR from the NG formulation suggests that such systems hold considerable potential for advancing targeted therapies, particularly for the treatment of chronic conditions such as cancer, where sustained drug exposure is critical for therapeutic success.

### 3.4. NGs Internalization into TC Cell Lines

We then tested the ability of the Fmoc-FF NGs to target TC cell lines. The Fmoc-FF NG is internalized in TC cells (K1 and CAL62).

**Figure 4:**
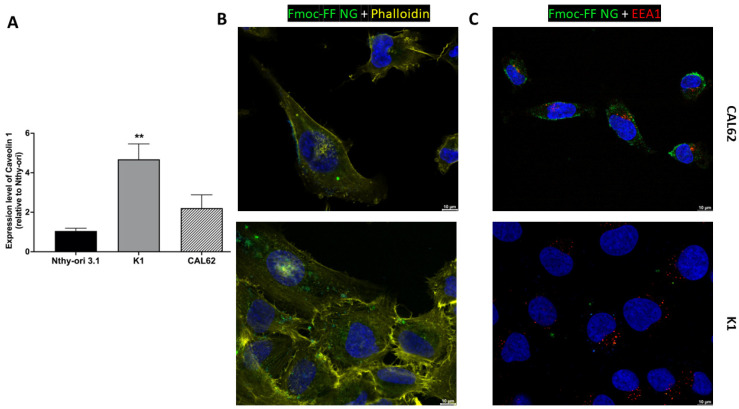
The internalization of Fmoc-FF NGs into TC cell lines. (**A**) The relative expression of Caveolin 1 in K1 and CAL62 cell lines compared to a non-transformed epithelial thyroid cell line (Nthy-ori 3.1) was evaluated by q-RT-PCR and calculated using the formula 2^−ΔΔCt^. *p*-value < 0.01 (**). Immunofluorescence analysis of CAL62 (upper panels) and K1 (lower panels) after 4 h of incubation with FITC-filled NGs. (**B**) Merge signals of Hoechst (blue, nuclei), Phalloidin (yellow, cytoskeleton), and FITC-filled NGs. (**C**) Merge signal of Hoescht (blue, nuclei), EEA1 antibodies (red, early endosome), and FITC-filled NGs. Magnification: 63×. Scale bar: 10 µm.

Figure 4B and Figure S4 in Supplementary Materials show that FITC-filled NGs localize mainly to the cytoplasm of K1 and CAL62 cells after 4 h of incubation, without localizing to early endosomes (Figure 4C and Supplementary Materials, Figure S5). This suggests that the Fmoc-FF NGs release the contents into the cell cytoplasm and that these are not rapidly degraded, ensuring intracellular stability during release.

Since we have previously shown that caveolins are primarily responsible for the NG intracellular uptake in the triple-negative breast cancer cell line MDA-MB-231 [41,44], we investigated the expression of Caveolin 1 in the TC cell lines used. As shown in Figure 4A, Caveolin 1 is highly expressed in TC cells (K1 and CAL62) compared to a non-transformed epithelial thyroid cell line, Nthy-ori 3.1, suggesting that caveolins may play a role in the internalization process.

### 3.5. Fmoc-FF Nanogels Reduces Uptake Kinetics of DOX and CUR

**Figure 5:**
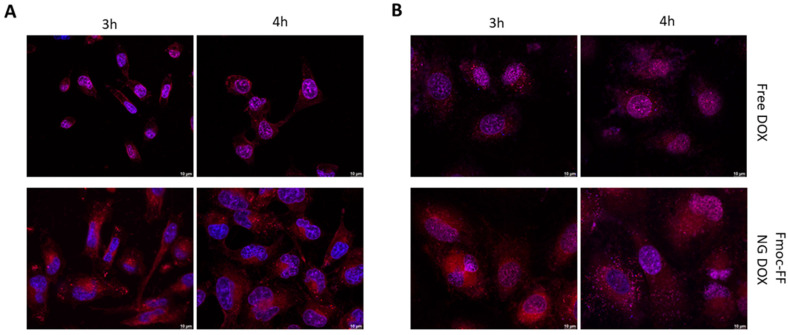
Immunofluorescence analysis of (**A**) CAL62 and (**B**) K1 at different times of incubation with free DOX (upper panels) or DOX-filled NGs (lower panels). Blue signals represent the nuclei staining (Hoechst). Red signals represent free and DOX-filled NGs. Magnification: 63×. Scale bar: 10 µm.

Then, we endeavored to assess Fmoc-FF NGs’ capacity to transport CUR and DOX in TC cells. As shown in Figure 5, DOX-filled NGs clearly reduced the rate of the nuclear internalization of DOX in both CAL62 (Figure 5A and Supplementary Materials, Figure S6) and in K1 (Figure 5B and Supplementary Materials, Figure S7) cell lines, when compared to free DOX (upper panels). Indeed, free DOX was already internalized in the nucleus after 3 h of incubation, whereas the DOX loaded through the Fmoc-FF NGs after 4 h of incubation clearly remained in the cytoplasm.

**Figure 6:**
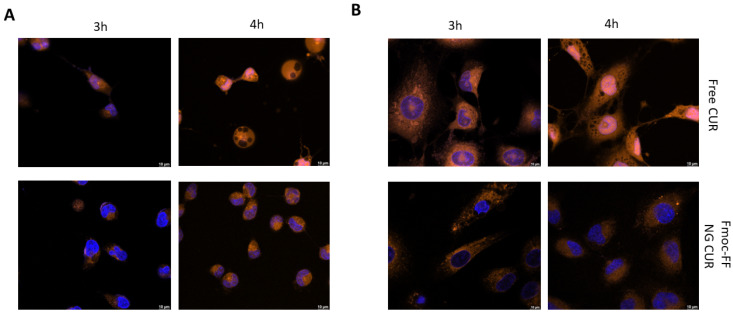
Immunofluorescence analysis of (**A**) CAL62 and (**B**) K1 at different times of incubation with free CUR (upper panels) or CUR-filled NGs (lower panels). Blue signals represent the nuclei staining (Hoechst). Orange signals represent free and CUR-filled NGs. Magnification: 63×. Scale bar: 10 µm.

The same experiments were repeated using CUR-filled NGs. As can be observed from Figure 6, the delaying effect of the Fmoc-FF NG when determining the internalization of CUR into the nucleus is even more striking than that of DOX. Whereas in CAL62, CUR carried by the Fmoc-FF NGs began to enter the nucleus after 4 h of incubation (Figure 6A and Supplementary Materials, Figure S8); in K1 cells treated with CUR-loaded Fmoc-FF NGs, after 4 h of incubation, 80% remained in the cytoplasm; CUR in a free form was almost completely nuclear (Figure 6B and Supplementary Materials, Figure S9).

### 3.6. Fmoc-FF Nanogels Are Able to Deliver DOX and CUR in TC Cell Lines

**Figure 7:**
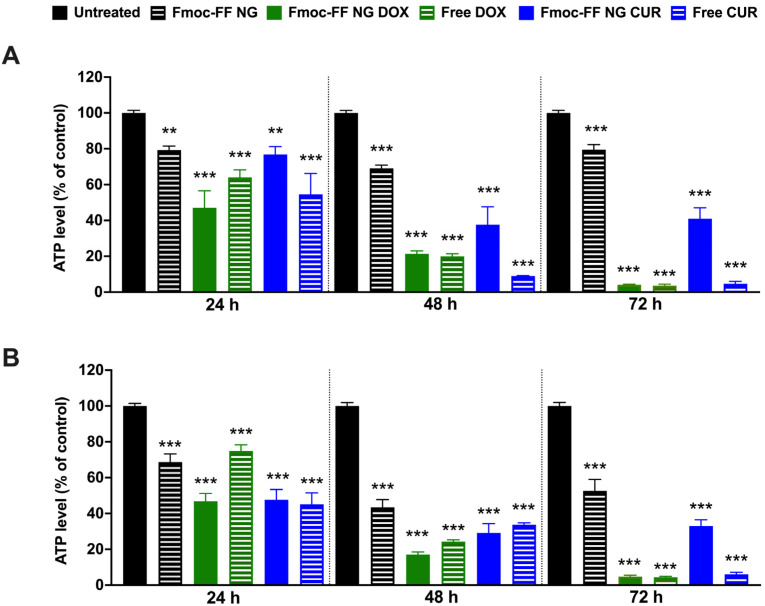
ATP levels of (**A**) K1 and (**B**) CAL62 untreated (black bars), or treated with Fmoc-FF NGs (black dashed bars), DOX-filled NGs (green bars), free DOX (green dashed bars), CUR-filled NGs (blue bars), and free CUR (blue dashed bars) at different incubation times. Statistical analyses were performed with the untreated TC cell lines at 100% viability. *p*-value < 0.01 (**), *p*-value < 0.001 (***), determined by one-way ANOVA. Error bars represent the standard error of the mean (SEM) from independent experiments.

We then evaluated the effects of DOX- and CUR-filled NGs on TC cell viability in K1 and CAL62 cells compared to free agents. Treatment with DOX-filled Fmoc-FF NGs significantly reduced K1 (Figure 7A) and CAL62 (Figure 7B) cell viability, similarly to the effect of free DOX, after 48 h of treatment. In contrast, CUR-filled NGs significantly reduced the viability of the tested cells but with lower efficiency than free CUR. In both cases, NGs at the conditions tested reduced the viability of the two cell lines at 24 and 48 h, while at 72 h, this effect appeared to be partially rescued (Figure 7). These findings are consistent with what was previously reported in the MDA-MB-231 cell line [52].

## 4. Discussion

Nanogels (NGs) are nanoparticles with a size suitable for intravenous injection and an internal structure resembling hydrogel matrix, in which fibrillary networks are capable of encapsulating a high water content [45]. Due to these unique characteristics, NGs can efficiently encapsulate both hydrophilic and hydrophobic molecules.

This study aims to develop novel therapeutic approaches for thyroid cancer, focusing on the potential of Fmoc-FF-based NGs as versatile scaffolds for the efficient delivery of various molecules into TC cells. To explore this, we selected two different classes of therapeutic agents: the hydrophilic drug DOX and the hydrophobic compound CUR.

Our findings demonstrate the ability of Fmoc-FF NGs to internalize into TC cells. These data suggest the need for a novel approach to targeted drug delivery in TC. Indeed, the internalization of Fmoc-FF NGs into TC cells may play a crucial role in their therapeutic efficacy. Our results indicate that Fmoc-FF NGs bypass early endosomes and localize to the cytoplasm of CAL62 and K1 cells within a short incubation period. This efficient uptake into cancer cells is likely facilitated by a high expression of Caveolin 1, suggesting the potential involvement of caveolins in the internalization process [52]. Interestingly, Zhang et al. observed high Caveolin 1 expression in papillary thyroid cancer [59]. Moreover, the cytoplasmic localization of FITC-loaded Fmoc-FF NGs implies that these NGs release their cargo directly into the cellular cytoplasm, which may contribute to their stability and sustained therapeutic effect [60].

Our study also highlights the versatility of Fmoc-FF NGs in delivering a variety of agents into TC cells. Specifically, Fmoc-FF NGs effectively deliver both DOX and CUR into CAL62 and K1 cells, delaying nuclear internalization compared to free drugs. Notably, the delayed nuclear internalization of CUR-filled NGs is particularly striking, suggesting the prolonged retention of CUR in the cytoplasm. This enhanced retention could improve the therapeutic efficacy of CUR by extending its exposure to tumor cells, thereby increasing its cytotoxic effect.

These findings suggest that Fmoc-FF NGs could effectively deliver a range of therapeutic or also imaging agents into TC cells, potentially improving both therapeutic and diagnostic outcomes for TC patients.

The development of effective drug delivery systems for TC therapy faces several challenges, including limited drug penetration into tumor tissues and systemic toxicity [61,62]. Fmoc-FF NGs offer several advantages in addressing these challenges. First, by bypassing early endosomes and releasing their cargo directly into the cytoplasm, they could facilitate more efficient drug transport within cells, thereby maximizing therapeutic effects. Secondly, the prolonged release of drugs from Fmoc-FF NGs may enhance drug retention in tumor tissues, boosting interactions with cancer cells while minimizing side effects. From a diagnostic standpoint, the release properties of Fmoc-FF NGs may enable the development of novel contrast agents with reduced radiotracer dosages [35]. Additionally, the biocompatibility of Fmoc-FF NGs lowers the risk of systemic toxicity, making them suitable for clinical translation [55]. By harnessing the special qualities of Fmoc-FF NGs, we can likely overcome the current restrictions on the delivery of drugs and/or contrast agents and enhance the effectiveness of TC patient care [53].

Lastly, combined therapeutic approaches may be enabled by Fmoc-FF NGs adaptability in the delivery of various drug types, which might boost therapeutic outcomes [63,64]. A limitation of this study is that, currently, we have not yet performed in vivo studies. In this context, the enhanced permeability and retention (EPR) effect is anticipated to play a pivotal role in facilitating the accumulation of the nanogels within tumor tissues. However, we plan to functionalize the surface of the nanogels to enhance their targeting capabilities. Additionally, we intend to conduct in vivo toxicity and biodistribution studies using murine models, which will be addressed in future research. It is important to note that the current literature predominantly emphasizes the development and application of polymeric nanogels, with comparatively less focus on peptide-based nanogels. Therefore, despite its limitations, the current study provides significant value and represents an innovative contribution for the development of innovative peptide-based nanogel delivery systems.

In conclusion, Fmoc-FF NGs could represent a promising platform for improving the treatment and diagnosis of thyroid cancer, particularly for cases that are resistant to conventional therapies. The continued research and development of nanomedicines have great potential for revolutionizing cancer treatment, providing more effective and less toxic alternatives to current therapies. Future studies should focus on further exploring the clinical applications of these NGs, as well as investigating their long-term safety and efficacy in in vivo models, ultimately paving the way for their successful translation into clinical practice.

## Figures and Tables

**Table 1:** Structural characterization of Fmoc-FF empty and nanogels filled with DOX and CUR drugs.

System	Mean Diameter (nm) ± S.D.	PDI	ζ mV ± S.D.	DLC%	ER%
Fmoc-FF NGs	174 ± 82	0.176	−24.0 ± 0.1	-	-
DOX-filled NGs	241 ± 72	0.183	−10.6 ± 0.4	13.7%	63%
CUR-filled NGs	204 ± 80	0.167	−34.6 ± 1.2	30.1%	90.8%

S.D.: standard deviations; PDI: polydispersity index; ζ: zeta potential; DLC: drug loading content; ER: encapsulation ratio.

## Data Availability

The datasets generated during and/or analysed during the current study are available from the corresponding author on reasonable request (giovanni.smaldone@synlab.it).

## Supplementary Materials

The following supporting information can be downloaded at https://www.mdpi.com/article/10.3390/pharmaceutics17020263/s1. Figure S1: FT-IR spectra of empty and CUR-filled HGs and NGs; Figure S2: Size distribution of particles using nanoparticle tracking analysis (NTA); Figure S3: Syringeability tests, DLS profile after extrusion and plot of viscosity of empty and filled NGs; Figure S4: Hoescht, FITC loaded FmocFF-NG and Phalloidin signals of immunofluorescence analysis; Figure S5: Hoescht, FITC loaded FmocFF-NG and EEA1 signals of immunofluorescence analysis; Figure S6: Hoescht and DOX signals of immunofluorescence analysis for CAL62 cells; Figure S7: Hoescht and DOX signals of immunofluorescence analysis for K1 cells; Figure S8: Hoescht and CUR signals of immunofluorescence analysis for CAL62 cells; Figure S9: Hoescht and CUR signals of immunofluorescence analysis for K1 cells.

## Abbreviations

The following abbreviations are used in this manuscript: (CUR) curcumin; (DLC) drug loading content; (DLS) dynamic light scattering; (DMEM) Dulbecco’s Modified Eagle Mediums (DMSO) dimethyl sulfoxide; (DOX) doxorubicin; (ECACC) European Collection of Authenticated Cell Cultures; (EMT) epithelial-to-mesenchymal transition; (EPR) enhanced permeability and retention; (ER) encapsulation ratio; (FBS) fetal bovine serum; (FITC) fluorescein-isothiocyanate; (FT-IR) Fourier transform infrared spectroscopy; (LVR) linear viscoelastic region; (NGs) nanogels; (NPs) nanoparticles; (NTA) nanoparticle tracking analysis; (PBS) L phosphate buffer solution; (PDI) polydispersity index; (RH) hydrodynamic radii; (SEC) size exclusion chromatography; (SEM) scanning electron microscopy; (TC) thyroid cancer.
