# Supplementary material for: Fmoc-FF Nanogel-Mediated Delivery of Doxorubicin and Curcumin in Thyroid Cancer Cells
Source: Pharmaceutics. 2025 Feb 17;17(2):263. doi: 10.3390/pharmaceutics17020263 (PMC11858838; doi:10.3390/pharmaceutics17020263)
Supplement: Supplementary file 1 [file pharmaceutics-17-00263-s001.zip › pharmaceutics-3456563-supplementary.pdf]

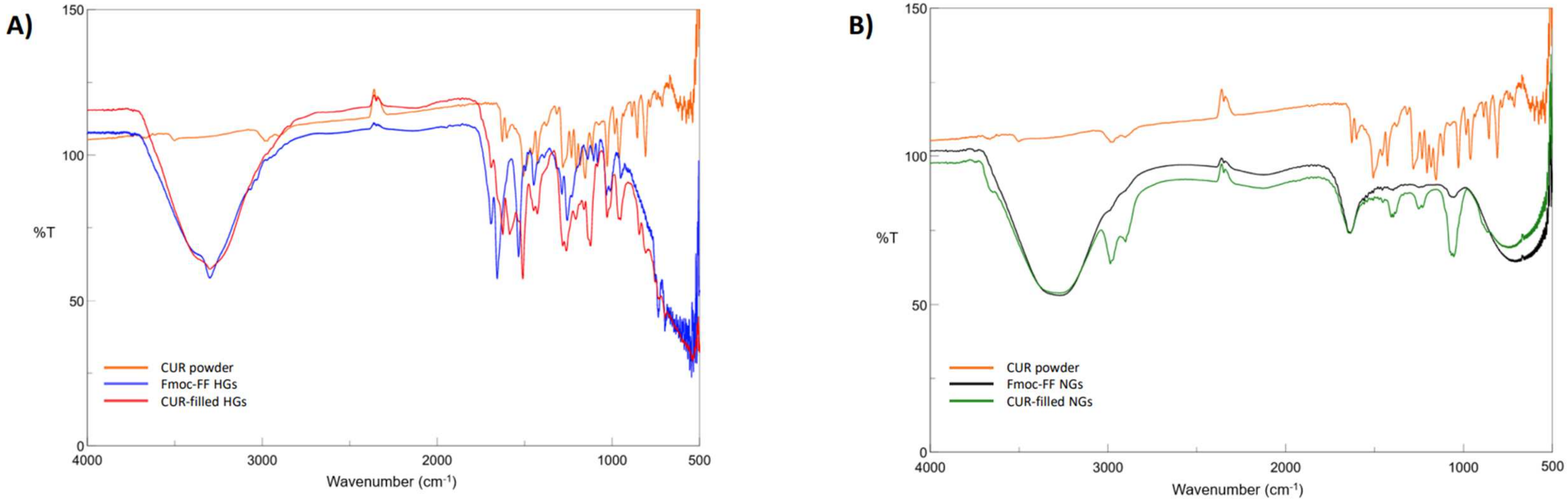

Supplementary Figure S1. Secondary structure analysis of Fmoc-FF hydrogels and nanogels. **(A)** Comparison between FT-IR spectra in 4000-400 cm^{-1} range of curcumin powder and empty and CUR-filled hydrogels; and of **(B)** curcumin powder and empty and CUR-filled nanogels;

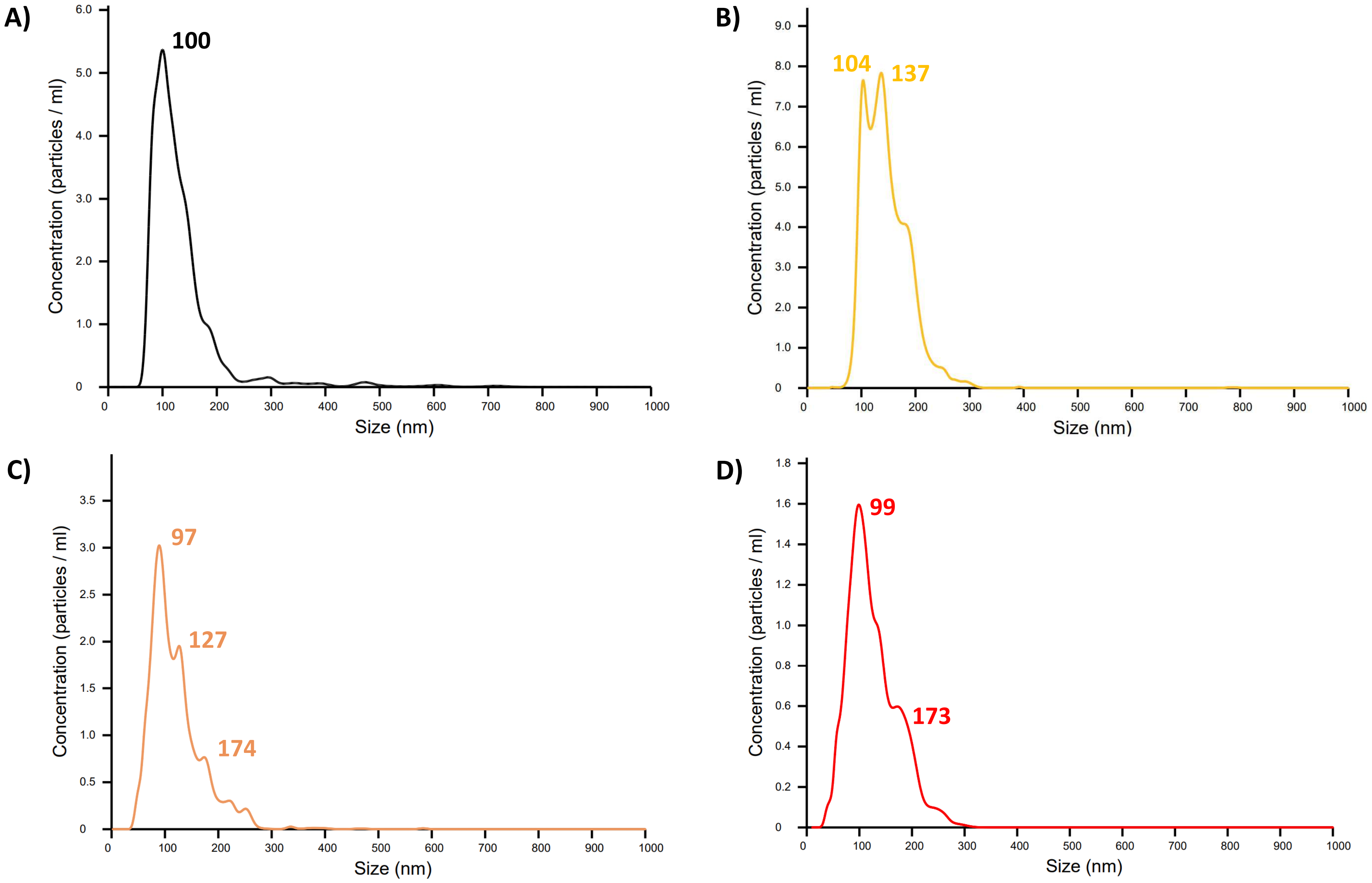

Supplementary Figure S2. Size distribution of particles using nanoparticle tracking analysis (NTA) for **(A)** empty Fmoc-FF NGs; **(B)** FITC-filled NGs; **(C)** CUR-filled NGs; **(D)** DOX-filled NGs.

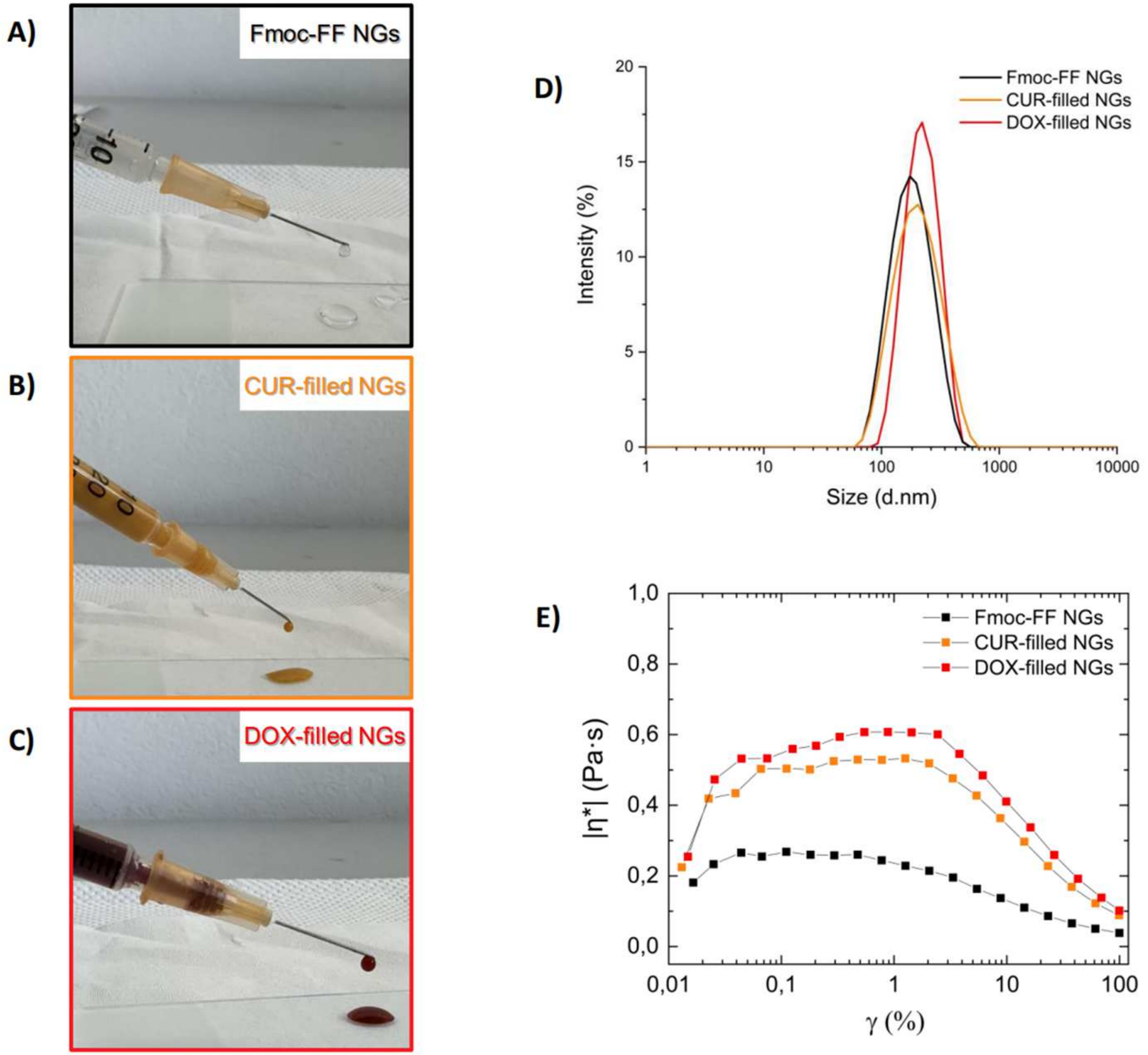

Supplementary Figure S3. Syringeability test of **(A)** empty Fmoc-FF NGs and of **(B)** CUR-filled NGs and **(C)** DOX-filled NGs; **(D)** DLS profiles after extrusion from syringe; **(E)** plot of viscosity over shear rate for different NG systems.

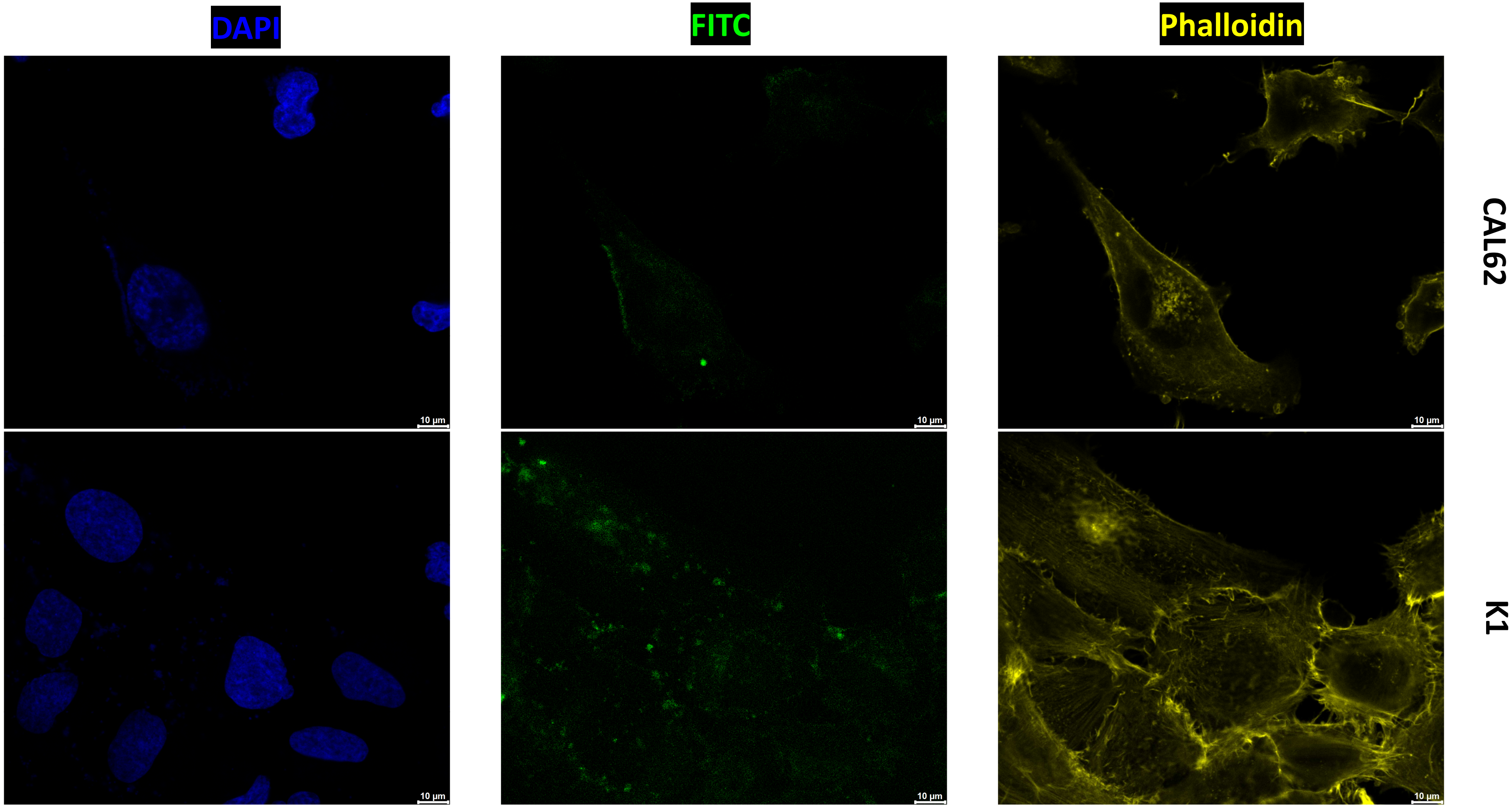

Supplementary Figure S4. Hoescht (left panels), FITC-filled NGs (middle panels) and Phalloidin (right panels) signals of immunofluorescence analysis reported in Figure 2B. CAL62 (upper panels) and K1 (lower panels). Magnification 63×. Scale bars 10 μm.

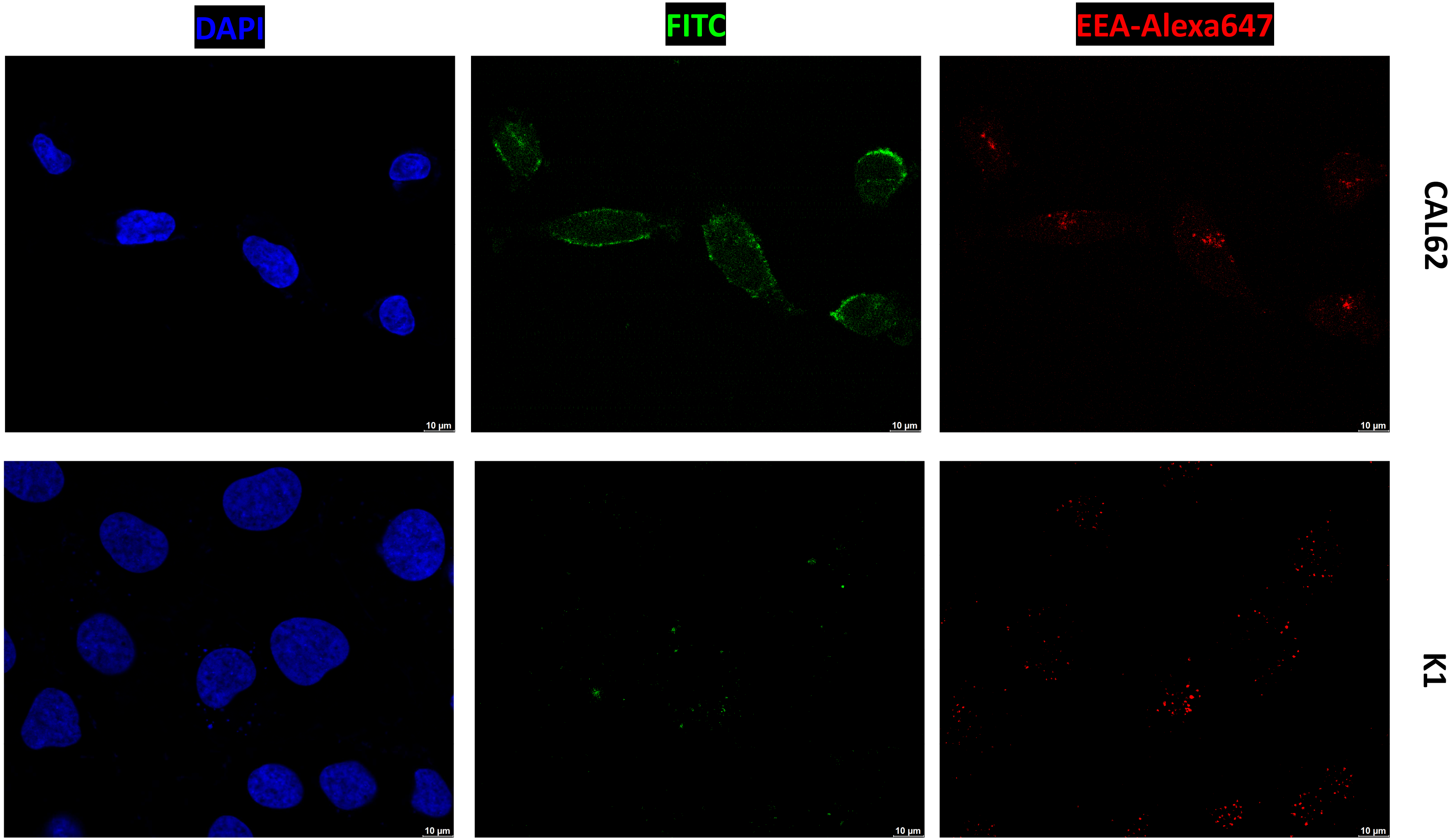

Supplementary Figure S5. Hoescht (left panels), FITC-filled NGs (middle panels) and EEA1 (right panels) signals of immunofluorescence analysis reported in Figure 2B. CAL62 (upper panels) and K1 (lower panels). Magnification 63×. Scale bars 10 µm.

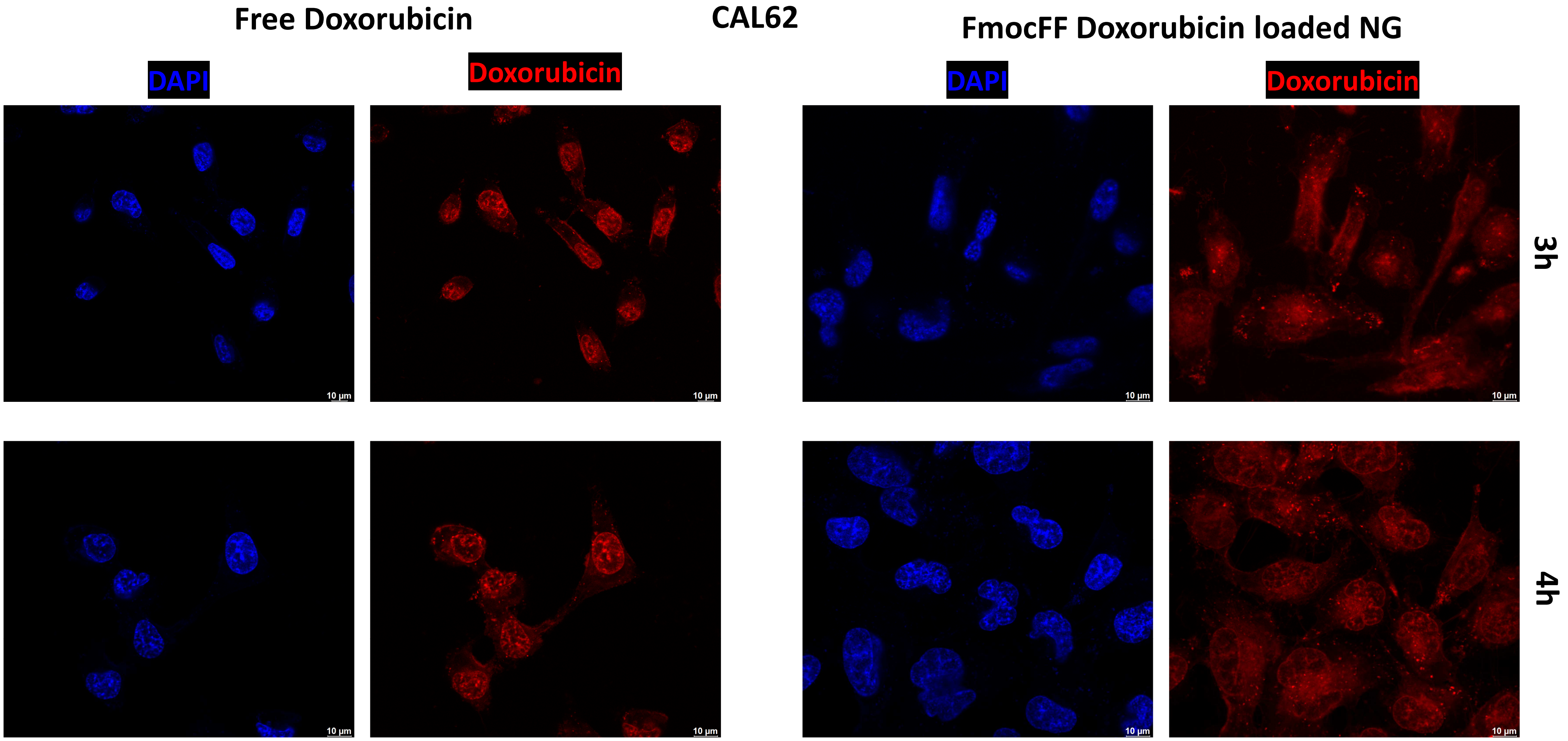

Supplementary Figure S6. Hoescht and doxorubicin signals of immunofluorescence analysis reported in Figure 5A . Magnification 63×. Scale bars 10 µm.

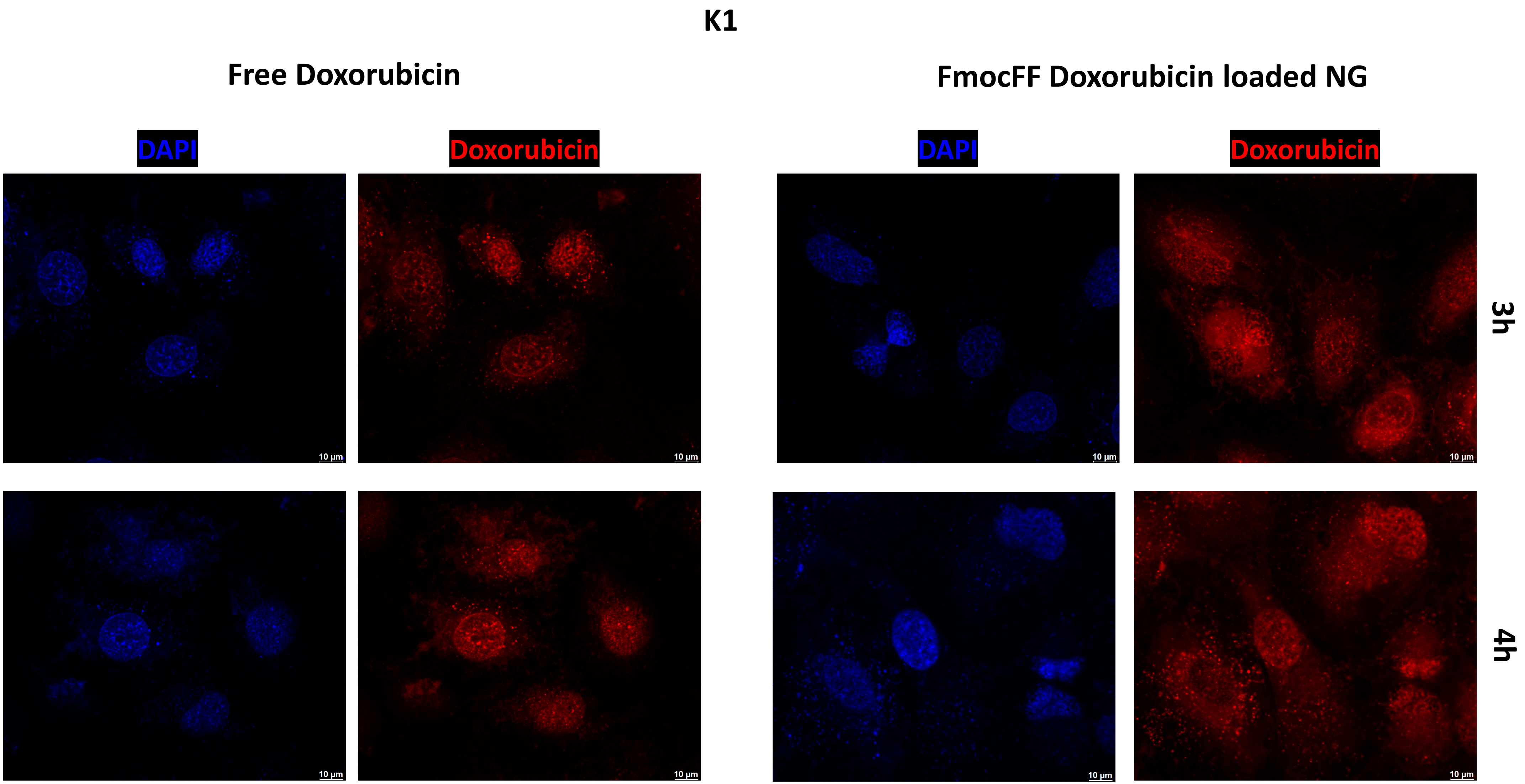

Supplementary Figure S7. Hoescht and doxorubicin signals of immunofluorescence analysis reported in Figure 5B. Magnification 63×. Scale bars 10 µm.

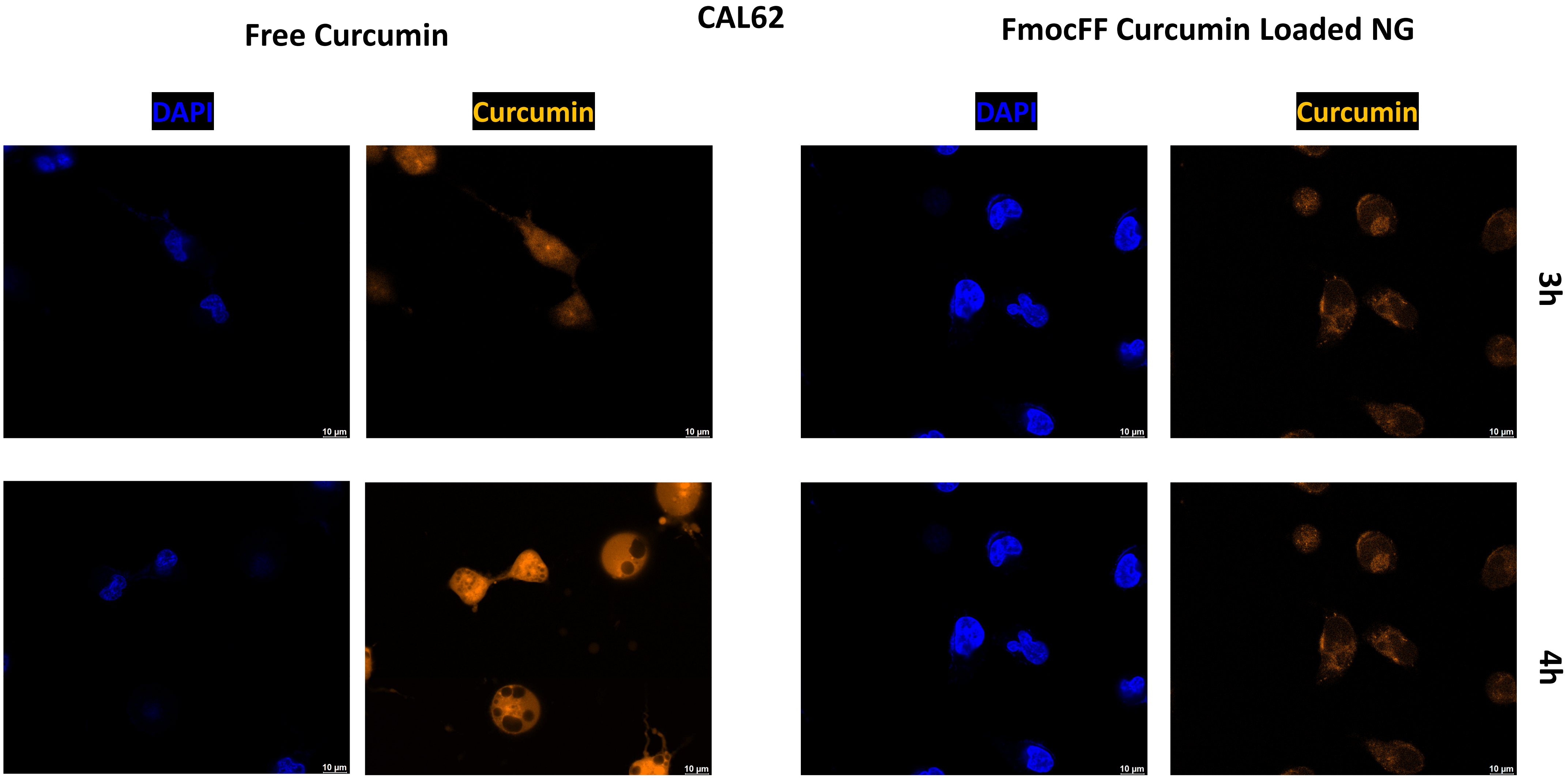

Supplementary Figure S8. Hoescht and curcumin signals of immunofluorescence analysis reported in Figure 6A. Magnification 63×. Scale bars 10 µm.

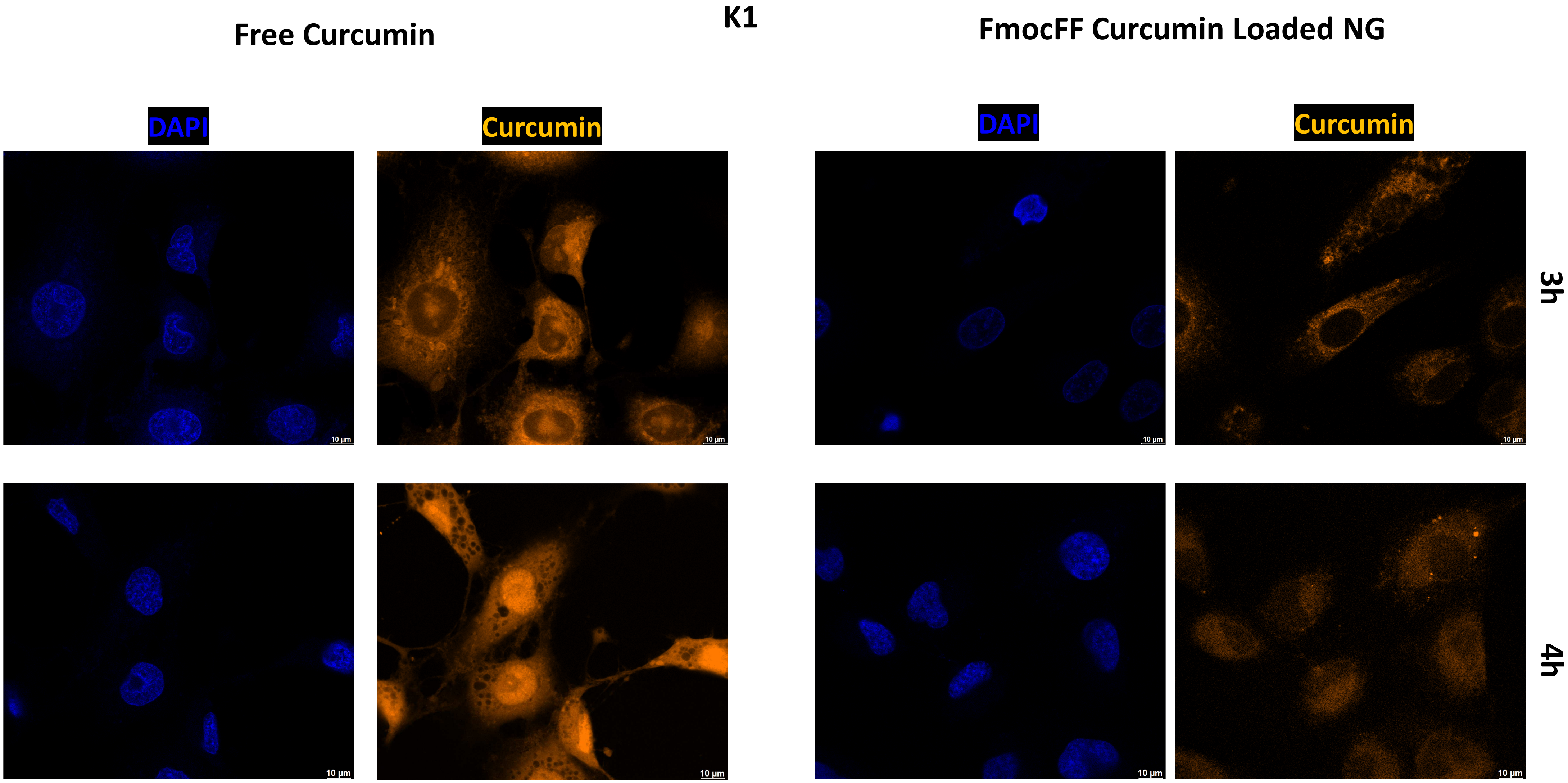

Supplementary Figure S9. Hoescht and curcumin signals of immunofluorescence analysis reported in Figure 6B. Magnification 63×. Scale bars 10 µm.
